# Developing Active Modified Starch-Based Films Incorporated with Ultrasound-Assisted Muña (*Minthostachys mollis*) Essential Oil Nanoemulsions

**DOI:** 10.3390/polym18010023

**Published:** 2025-12-22

**Authors:** José Antonio Flores-Bao, Luis Jaime Pérez-Córdoba, Patricia Martínez-Tapia, Fiorela Peña-Carrasco, Paulo José do Amaral Sobral, Izabel Freitas Moraes, Carmen Velezmoro-Sánchez

**Affiliations:** 1 Programa de Doctorado en Ciencia de Alimentos, Universidad Nacional Agraria La Molina, Av. La Molina s/n La Molina, Lima 15024, Peru; joseantoniofloresbao@gmail.com; 2 Departamento de Ingeniería de Alimentos, Facultad de Industrias Alimentarias, Universidad Nacional Agraria La Molina, Av. La Molina s/n La Molina, Lima 15024, Peru; ljperezcordoba@gmail.com; 3 Programa de Doctorado en Nutrición, Universidad Nacional Agraria La Molina, Av. La Molina s/n, La Molina, Lima 15024, Peru; 20240281@lamolina.edu.pe; 4 Department of Food Engineering, School of Animal Sciences and Food Engineering, University of São Paulo, Pirassununga 13635-900, SP, Brazil; pjsobral@usp.br (P.J.d.A.S.); bel@usp.br (I.F.M.); 5 Food Research Center (FoRC), University of São Paulo, Rua do Lago, 250, Semi-Industrial Building, Block C, São Paulo 05508-080, SP, Brazil

**Keywords:** *Minthostachys mollis*, tunta starch, I-optimal design, essential oil nanoemulsions, active films

## Abstract

In this study, an I-optimal design was used to select an optimal muña essential oil nanoemulsion (MEO-NE) for application in active starch-based films. Four independent variables were used to optimize the process: emulsifier concentration (X_1_) (% *w*/*w*), sonication time (X_2_) (min), essential oil concentration (X_3_) (% *w*/*w*), and emulsifier type (X_4_) (Tween 80 or sapote gum). Results revealed that MEO-NE containing 5.24% of MEO, 6% Tween^®^ 80, and 9 min of ultrasound treatment exhibited a small droplet size (Y_1_) (48.6 nm), moderate ζ-potential (Y_2_) (−15 mV), and DPPH inhibition (Y_3_) (95.6%). Starch-based films were incorporated with optimized MEO-NE at 5% (F_1_) and 10% (F_2_) and compared with control films (F_0_). F_1_ and F_2_ exhibited lower moisture content, water solubility, and water vapor permeability than F_0_; however, their contact angles were higher. The addition of MEO-NE into the polymeric matrix increased the stiffness of F_1_ and F_2_; however, the elongation at yield was slightly lower than that of F_0_, resulting in less stretchable composite films. All films were disintegrated by more than 90% after 5 days of burial under composting conditions. The incorporation of MEO-NE into composite films significantly enhanced their properties, suggesting their potential use as eco-friendly packaging.

## 1. Introduction

Nowadays, essential oil nanoemulsions (oil/water systems) are used to disperse aromatic substances with antioxidant and antimicrobial activities [1,2,3,4]. Among these substances, the essential oils of aromatic and medicinal plants stand out [5], such as those of *Minthostachys mollis,* a species native to the Andean region and commonly known as muña in Peru. Different researchers have identified up to 40 components, with menthone, carvacrol, thymol, and eucalyptol as the most abundant; however, these findings depend on where the plants grow and the extraction methods used, as reported by some authors [6,7]. On the other hand, the small droplets (up to 500 nm) obtained in a nanoemulsion do not require a lipophilic balance among their components; emulsifiers are still necessary to improve their stability over time [8]. Recently, the use of polymers, such as proteins, gums, or modified starches, among others, has gained importance for stabilizing emulsions without the need for synthetic stabilizers [1,8,9]. In this regard, among the polysaccharides used to stabilize emulsions, Arabic gum has proven to be a suitable natural emulsifier; however, concentrations greater than 5% are required to achieve optimal stability [9]. A less-studied polysaccharide is the gum from the *Capparis scabrida* H.B.K. tree, known in Peru as Sapote, which is obtained by exuding the bark. This gum has been used to create a coating for bananas, extending their shelf life [10], and could also serve as an emulsifier.

Nanoemulsions can be obtained by various methods, including high-pressure and ultrasound, which are considered high-energy methods for their formation [11]. Different authors have investigated the power and application time of ultrasound to produce stable emulsions, as demonstrated in the cases of curcumin/soybean essential oil and orange/soybean essential oil [12]. Likewise, the time and temperature parameters have been optimized to obtain nanoemulsions with cinnamon essential oil and Tween 80 at different percentages [13] or with different emulsifiers [14].

To determine the optimal parameters for a process, such as obtaining nanoemulsions, an I-optimal design can be used, including both categorical and numerical factors, and response surface modeling can be applied to minimize average prediction variance across the entire design space. In contrast, D-optimal designs focus on parameter estimation, whereas I-optimal designs are best for accurate predictions across a large portion of the experimental region [15].

Among the applications of nanoemulsions, their use in food has been investigated as a vehicle for lipid-based antimicrobial and antioxidant additives, which can be incorporated into aqueous media [11]. Likewise, the inclusion of essential oils in biodegradable films has led to the term ‘active packaging’ due to the functional properties they provide [16,17]. These biodegradable films are based on various biopolymers, including complex carbohydrates (such as starch, gum, pectin, and chitosan) and proteins (derived from whey, soy, quinoa, and other sources). These can be presented alone or in mixtures. By incorporating emulsified essential oils, they can transmit the functional properties of these oils to the foods they are protecting.

Potato starch has been widely studied but has received less attention due to its grain size, amylose content, and the high viscosity of the gels it forms. Some researchers, however, have developed edible or biodegradable active films based on potato starch [18,19,20,21]. Although the properties of the films obtained have been affected by the addition of essential oils, nanoemulsions, or starch modification, further research is needed to investigate the extent of these modifications and the interactions among film components, with a focus on specific applications tailored to targeted food. Due to the limited inclusion of chemical groups in starch molecules, which could cause long-term health harm, chemical modifications are losing popularity. Consequently, physical changes are receiving increased research attention. A modification using continuous natural freeze–thaw processes [22] demonstrated the potential to reduce the viscosity of potato starch gels, which contributed to the production of disintegrable films reinforced with tara gum and starch nanocrystals [23].

The incorporation of ultrasound-assisted muña (*Mynthostachys mollis*) essential oil nanoemulsions (MEO-NE) into active films based on naturally modified potato starch, prepared through freeze–thaw processing in a blend with chitosan, was investigated. MEO-NEs were prepared using Tween^®^ 80 and sapote gum (SG); both types of NE were studied under an I-optimal design to obtain a stable nanoemulsion with high antioxidant activity. To our knowledge, there are no studies on the formation of nanoemulsions stabilized with SG, nor the development of active starch-based films incorporating muña essential oil nanoemulsions.

## 2. Materials and Methods

### 2.1. Materials

Muña (*Minthostachys mollis*) essential oil (MEO) (23.77% menthone, 22.67% pulegone) was purchased from Essential Oils Peru (Lima, Peru). Tunta starch (TS) was previously isolated and characterized by Martínez et al. [22]. Sapote gum (SG) was provided by a local farmer in Almirante Miguel Grau, a small town (Cura Mori, Piura, Peru), and it was purified before use according to Vélez-Erazo et al. [10]. The evaluated plant material Solanum curtilobum was accessed in Peru in accordance with access contract No. 003-2025-MIDAGRI-INIA/DGIA and Directoral Resolution No. 003-2025-INIA-DGIA. Tween^®^ 80, glycerol, and chitosan (medium molecular-weight, deacetylation degree: ≥75%, and viscosity: 200–800 cP) were purchased from Sigma-Aldrich^®^ (Darmstadt, Germany). Additionally, 2,2-diphenyl-1-picrylhydrazyl (DPPH) was purchased from Sigma-Aldrich^®^ (China), and anhydrous ethanol was obtained from Merck^®^ (Darmstadt, Germany). Other chemical reagents used in this research were of analytical grade.

### 2.2. Ultrasound-Assisted MEO-NE Preparation

The ultrasound-assisted nanoemulsions were prepared using MEO as the disperse phase and deionized water as the continuous phase, following the method of Chu et al. [24] with minor modifications. Firstly, Tween^®^ 80 or Sapote gum (6% and 10% *w*/*w*) was dissolved in deionized water using a magnetic stirrer at 800 rpm and room temperature for 30 min. Then, MEO (3% and 6% *w*/*w*) was added to the solutions, and the coarse emulsions were prepared by homogenization at 12,000 rpm for 4 min using a high-speed homogenizer (T25 Digital Ultra-Turrax^®^, IKA, Staufen, Germany). After that, the obtained coarse emulsions were subjected to ultrasonic emulsification for different sonication times (9 and 15 min) using a sonicator (VC 505, Sonics, Dallas, TX, USA) with a power output of 500 W and an amplitude of 60%. A 0.5-pulg sonicator probe was used, and the heat produced during treatment was dissipated using an ice-water bath.

### 2.3. Optimization of MEO-NE Using I-Optimal Design

The formulation (6 to 10% emulsifier concentration, 3 to 6% essential oil concentration, and type of emulsifier, Tween^®^ 80 or sapote gum) and condition (sonication time of 9 to 15 min) that yielded the optimum MEO-NE were determined using the I-optimal design. This design was applied to investigate the effects of independent variables: emulsifier concentration (X_1_), sonication time (X_2_), essential oil concentration (X_3_), and type of emulsifier (X_4_) on the responses: droplet size (Y_1_), ζ-potential (Y_2_), and DPPH inhibition (Y_3_) for each run of MEO-NE. The levels of independent variables used in the I-optimal design are depicted in Table 1.

An I-optimal experimental design was generated, consisting of 24 experimental runs with three replicates (Table 2). Each factor level combination was randomized. A sequential model-fitting process was then performed using three tests (sequential model sum of squares, lack-of-fit tests, and model summary statistics) to evaluate the models’ competency. The results showed that the best-fitting model was a second-order polynomial model with main effects, two-factor interactions, and quadratic terms. Quadratic terms capture the curvature in the relationship between the response (Droplet size, ζ-potential, DPPH inhibition) and the experimental variables. A second-order polynomial equation was applied to indicate the responses that were a function of the independent variables (Equation (1)).

(1) $$Y_{i}=a_{0}+a_{1}X_{1}+a_{2}X_{2}+\cdots +a_{n}X_{n}+\sum a_{ij}X_{i}X_{j}+\sum a_{ii}X_{i}^{2}$$

where $Y_{i}$ is the response function (*i* = 1, 2, and 3), a_0_ is a constant, and the regression coefficients in Equation (1) are a_1_ to a_ii_. The independent variables are represented as linear ( $X_{i}$ ), quadratic ( $X_{i}^{2}$ ), and interaction terms ( $X_{i}X_{j}$ ) (*i*, *j* = 1, 2, 3, and 4), respectively.

### 2.4. Characterization of MEO-NE

#### 2.4.1. Droplet Size, Polydispersity Index (*PDI*), and ζ-Potential

The droplet size, *PDI*, and ζ-potential of nanoemulsions (NEs) were measured using a size analyzer (Litesizer 500, Anton Paar, Graz, Austria) and Milli-Q water, following the method of Chu et al. [24]. The ζ-potential was measured using 0.1 mM KCl to dilute the emulsions to a 1:100 ratio, as described by Shamsara et al. [25]. All analyses were conducted at 25 °C in triplicate.

#### 2.4.2. *DPPH^●^* Method

The antioxidant activity of the nanoemulsions was assessed using the *DPPH* free radical scavenging assay, as described by Pérez-Córdoba et al. [26]. Briefly, 100 μL of each MEO-NE was added to 3.9 mL of *DPPH^●^* methanolic solution (60 μM). After 60 min of incubation in the dark, absorbance was measured at 515 nm using a spectrophotometer (Genesys 10S UV-Vis, Thermo Scientific, Madison, WI, USA). The antioxidant activity, expressed as percentage *DPPH^●^* inhibition, was calculated using Equation (2):

(2) $${\mathrm{DPPH}}^{●}\left(\%\;\mathrm{inhibition}\right)=\left(\frac{A_{\mathrm{DPPH}}-A_{\mathrm{sample}}}{A_{\mathrm{DPPH}}}\right)\times 100$$

where *A_DPPH_* represents the absorbance of the *DPPH^●^* solution, and *A_sample_* denotes the absorbance of the sample.

#### 2.4.3. *ABTS^●+^* Method

The antioxidant activity of the nanoemulsions was assessed using the *ABTS^●^^+^* assay, as described by Pérez-Córdoba et al. [26]. Briefly, 300 μL of each MEO-NE was added to 2700 μL of *ABTS^●^^+^* solution, and the mixture was kept in the dark for 6 min. The absorbance was measured at 734 nm using a spectrophotometer (Genesys 10S UV-Vis, Thermo Scientific, Madison, WI, USA). The antioxidant activity, expressed as percentage *ABTS^●^^+^* inhibition, was calculated using Equation (3):

(3) $${\mathrm{ABTS}}^{●+}\left(\%\;\mathrm{inhibition}\right)=\left(\frac{A_{\mathrm{ABTS}}-A_{\mathrm{sample}}}{A_{\mathrm{ABTS}}}\right)\times 100$$

where *A_ABTS_* represents the absorbance of the *ABTS^●^^+^* solution, and *A_sample_* denotes the absorbance of the sample.

#### 2.4.4. *pH*

*pH* values of the NE were equilibrated at approximately 25 °C and determined by direct immersion of the potentiometer electrode (Basic20, CRISON, Barcelona, Spain), which had been previously calibrated with buffer solutions (pH 4.0, 7.0, and 9.0) according to the AOAC method 981.12/90 [27].

#### 2.4.5. Viscosity Measurements

The apparent viscosity of MEO-NE was measured using a hybrid rheometer (HR-3 Discovery, TA Instruments, New Castle, DE, USA) with a 40 mm parallel stainless-steel plate. The NEO was transferred to the platform, and the gap between the plate and the platform was adjusted to 1 mm. After equilibrating for 5 min at 25 °C, the viscosity was measured over a shear rate range of 1 s^−1^ to 100 s^−1^. The flow data were fitted to the Power Law and Herschel–Bulkley models using OriginPro 8 software (OriginLab Corporation, Northampton, MA, USA).

#### 2.4.6. Creaming Stability Analysis

For creaming index (*CI*) determination, 10 mL of each MEO-NE was transferred into a cylindrical test tube (inner diameter, 1 cm; height, 10 cm), tightly sealed with a rubber cap, and then stored at ~25 °C. During storage, phase separation occurred, with MEO-NE forming an optically thin cream layer at the top and a transparent (or turbid) serum layer at the bottom. The total height of the NEO (*H_E_*) and the height of the serum layer (*H_S_*) were measured as described by Liao et al. [28]. *CI* was determined as follows (Equation (4)):

(4) $$\mathrm{CI}\;\left(\%\right)=\left(\frac{H_{S}}{H_{E}}\right)\times 100$$

#### 2.4.7. Accelerated Stability Test

The physical stability of the MEO-NE was determined using an analytical centrifugal analyzer (LUMiSizer^®^, LUM GmbH, Berlin, Germany). This technique employs a centrifugation system to accelerate the onset of instability. Light (near-infrared and blue) passes through the sample, and the intensity of the transmitted light is detected as a function of time and position along the entire sample length. The experiment was performed by placing a 22 mm sample fill height in rectangular polycarbonate cells (r = 130 mm), which were exposed to centrifugal force at 4002 rpm for one hour at 25 °C, with an interval time of 10 s and a wavelength of 865 nm. The analyses were obtained in duplicate. Instability values were obtained directly from the SepView v.4.1 software (LUM GmbH, Berlin, Germany) and converted to stability as the percentage of light beam transmittance [29].

#### 2.4.8. Atomic Force Microscopy (*AFM*)

The optimal MEO-NE was analyzed by atomic force microscopy (*AFM*) (Solver next, NT-MDT, Moscow, Russia) to characterize droplet shape and surface morphology. The sample was diluted in ultrapure water (1:10 *v*/*v*), and a ~15 μL droplet was placed on a cover glass, which was then incubated at 25 °C for 24 h to allow the sample to adsorb to the surface. The analysis was carried out in intermittent-contact mode using the NSG01 probe at a scan rate of 0.7 Hz. In each test, a three-dimensional image of the optimized MEO-NE surface area (5 µm × 5 µm) and a two-dimensional image (2 µm × 2 µm) were obtained. Statistical parameters were calculated and analyzed using Nova Px 3.2.5 software (NT-MDT, Moscow, Russia), and imagen’s treatment was done with Image Analysis 3.2.5 software (NT-MDT, Moscow, Russia) [30].

### 2.5. Film Preparation

Active composite starch-based films were developed using the solvent-casting procedure as follows. The film-forming dispersion (FFD) was based on tunta starch (TS) and its blend with chitosan (Ch). On the one hand, Ch (2% *w*/*w*) was dispersed in an aqueous acetic acid solution (2% *v*/*w*) under magnetic stirring at 40 °C and 400 rpm for 24 h [23]. On the other hand, the starch dispersion was prepared with TS (4% *w*/*w*), heated to 90 °C for 30 min under magnetic stirring (600 rpm) until gelatinization, and then cooled to 50 °C. MEO-NE and glycerol (15 g/100 g polymers) (plasticizer) were added separately to the TS dispersion and the Ch solution with magnetic stirring until their incorporation. Both the TS dispersion and the Ch solution were mixed for 5 min at 14,000 rpm using an Ultra-Turrax^®^ T25 (IKA, Staufen, Germany). Then, the final blend FFD was degassed in a sonicator bath (DL 510 H Sonorex Digiplus, BANDELIN electronic GmbH & Co., Berlin, Germany) at 50 °C for 30 min. Finally, FFD was poured onto a polystyrene Petri plate (14 cm in diameter) and dried in a forced-air oven (MA035/5, Marconi, Piracicaba, SP, Brazil) at 35 °C for 24 h. Once dried, the films were removed from the plates and conditioned at room temperature in desiccators containing a saturated NaBr solution (58% relative humidity) for at least 5 days before characterization. Composite starch-based films were labeled as F_0_ (film without MEO-NE—Control), F_1_ (film loaded with MEO-NE to achieve 5 g MEO/100 g polymers), and F_2_ (film loaded with MEO-NE to achieve 10 g MEO/100 g polymers).

### 2.6. Film Characterization

#### 2.6.1. Film’s Appearance and Thickness

The appearance and homogeneity of composite films were evaluated by qualitative visual inspection. The thickness was determined at 10 random points on each sample surface using a digital micrometer (543–391, Mitutoyo, Kawasaki, Japan) with an accuracy of 0.001 mm [23]. The average value of these measurements was reported.

#### 2.6.2. Moisture Content (*MC*) and Solubility in Water (*SW*)

The composite films were cut into small discs (20 mm in diameter) to determine their moisture content (*MC*) and water solubility (*SW*). *MC* was evaluated by mass reduction of three film discs in a forced-air oven (Venticell 55, MMM, Munich, Germany) at 105 °C for 24 h. For *SW* measurement, three discs were weighed (m_0_) and immersed in distilled water (50 mL) under stirring in an orbital shaker (TOU-120, MRC, Holon, Israel) at 25 °C and 80 rpm for 24 h. Composite film samples were removed from the solution, dried in a forced-air oven (Venticell 55, MMM, Munich, Germany) at 105 °C for 24 h, and reweighted (m_f_) [23]. *SW* was calculated according to Equation (5):

(5) $$\mathrm{SW}\left(\%\right)=\left(\frac{m_{0}-m_{f}}{m_{0}}\right)\times 100\%$$

#### 2.6.3. Water Vapor Permeability (*WVP*)

*WVP* was determined using the method reported by Condés et al. [31]. Each composite film sample was sealed over a permeation cell containing silica gel (~0% RH), with a circular opening of 0.00317 m^2^, and stored in a desiccator at 25 °C. To maintain a 100% RH gradient across the film sample, distilled water was placed inside the desiccator. Water vapor transport was determined by measuring the weight gain of the permeation cell at steady state. Seven weight measurements were taken over 7 h, and the results were plotted as a function of time. The *WVP* was determined using Equation (6).

(6) $$\mathrm{WVP}=\left(\frac{W}{t}\right)\times \left(\frac{x}{A\;\Delta P}\right)$$

where *WVP* is water vapor permeability, *W*/*t* is the angular coefficient of the linear regression (g/s), *x* is the film thickness (m), *A* is the permeation area (0.0032 m^2^), and Δ*P* is the partial vapor pressure difference between the dry atmosphere and water (2642 Pa at 25 °C). The results were expressed as g·m^−1^·s^−1^·Pa^−1^, and three replicates per film were measured.

#### 2.6.4. Opacity

The opacity of the films was measured using a light transmission barrier assay, as described by Condés et al. [31]. Briefly, three film samples were cut into rectangular pieces and placed into the UV-Vis spectrophotometer cell (Genesys 10S UV-Vis, Thermo Scientific, Madison, WI, USA). The spectrum of each film was measured in transmittance mode (200–800 nm). The opacity was calculated using Equation (7), proposed by Qian et al. [32], and expressed in units of mm^−1^.

(7) $$\mathrm{Opacity}\;\left({\mathrm{mm}}^{-1}\right)={\mathrm{Abs}}_{\left(500\mathrm{nm}\right)}/\mathrm{film}\;\mathrm{thickness}\;\left(\mathrm{mm}\right)$$

#### 2.6.5. Contact Angle

Water contact angles were measured using an optical tensiometer (Theta Attension Lite, KVS Instruments, Finland). A drop of approximately five μL of ultrapure water was carefully placed on the film surface (6 cm^2^) using a microsyringe (Hamilton Gastight Syringes, Reno, NV, USA). Both sides of the water droplet were measured at room temperature. The results were given as the average of three determinations [33].

#### 2.6.6. Fourier Transform Infrared Spectroscopy (FTIR)

The FTIR spectra of composite films were obtained on a spectrometer (Spectrum-One, PerkinElmer, Waltham, MA, USA) using a DTGS detector and the ATR Smart iTX accessory. Each FTIR spectrum was recorded over 4000–550 cm^−1^, with a resolution of 4 cm^−1^ and 32 scans averaged. The Origin software was used to process the data.

#### 2.6.7. Mechanical Properties

The tensile strength (TS) and elongation at break (EB) of the composite films were determined using the method reported by Pérez-Córdoba et al. [23]. The test was performed using a texture analyzer machine (Model 5984, Instron, Norwood, MA, USA) equipped with a tensile grip probe. The films were cut into strips (7 cm × 1.5 cm), tested with a grip separation of 50 mm and a speed rate of 1 mm/s till breaking. For *TS* and *EB* measurements, at least 10 strips per treatment (n = 3) were measured, and Bluehill 3 software (Bluehill^®^, Instron, Norwood, MA, USA) was used to collect the data.

#### 2.6.8. Determination of Antioxidant Activity

Composite film samples (0.1 g) were immersed in 10 mL of a hydroalcoholic mixture (1:1, *v*/*v*) and kept under agitation at 80 rpm and 20 °C for 24 h to achieve the extraction of the bioactive compounds incorporated in MEO-NE. Antioxidant tests were performed in triplicate.

##### *DPPH^●^* Test

According to Pérez-Córdoba et al. [26], the solubilized composite film was centrifuged (4000 rpm, 30 min), and an aliquot (1.5 mL) was added to 1.5 mL of *DPPH^●^* radical solution (60 mM). Then, the mixture was kept in the dark for one hour. After this period, the absorbance was determined at 515 nm using a UV–Vis spectrophotometer. Antioxidant activity was calculated and expressed as a percentage of inhibition.

##### *ABTS^●+^* Test

This assay was carried out according to the method of Pérez-Córdoba et al. [26]. First, a solution containing *ABTS^●^^+^* radical (7 mM) and potassium persulfate (2.45 mM) was mixed (1:0.5) and kept in the dark for 16 h. Then, an aliquot of this solution was diluted with ethanol to obtain the *ABTS^●^^+^* working solution (absorbance = 0.70 ± 0.02 at 734 nm, measured with a UV–Vis spectrophotometer). 100 μL of solubilized composite film was centrifuged (4000 rpm, 30 min) and added to the *ABTS^●^^+^* working solution (900 μL), and the obtained mixture was kept in darkness for 6 min. Antioxidant activity was calculated and expressed as a percentage of inhibition.

#### 2.6.9. Disintegrability Test

An expanded polystyrene box (~5 L) was filled with compost from the Planta de Compostaje of the Universidad Nacional Agraria La Molina (Lima, Peru), which was used for the disintegrability test of the films according to Pérez-Córdoba et al. [23] and the Standard ISO 20200:2015 [34]. The films were cut into 2 cm discs (3 discs per film) and dried in a forced-air oven (Venticell 55, MMM, Munich, Germany) at 105 °C for 24 h until a constant weight was achieved. Once dried, the samples were entirely buried in the wet compost (10 cm deep) and left for 5 days under aerobic conditions. The box was maintained at 25 °C, with daily water additions to prevent evaporation and maintain a relative humidity of ~50%. The samples were carefully removed from the wet compost after 5 days and gently washed with distilled water to remove the compost adhering to the individual meshes and the film surface. The samples were then dried at 105 °C for 24 h and reweighed. The percentage of disintegration was calculated using Equation (8) proposed by Goswaim and Maiti [35].

(8) $$D\;\left(\%\right)=\left[\left(W_{0}-W_{t}\right)/W_{0}\right]\times 100$$

where *D* is the disintegrability, *W*_0_ is the initial weight of the dry film, and *W_t_* is the weight of the degraded film at 5 days.

### 2.7. Statistical Analysis

Design-Expert software (version 12.0.3.0, Stat-Ease Inc., Minneapolis, MN, USA) was used to analyze experimental data. Multiple regression analysis was performed to fit the model to the experimental data, yielding an equation. The adequacy of the developed models was evaluated using the F-value, the coefficient of determination (R^2^), and the lack-of-fit test. Minimization and maximization of polynomials thus fitted were performed using the numerical optimization technique of this statistical package. To compare the treatments used to obtain active films (F_0_, F_1_, and F_2_), one-way analysis of variance (ANOVA) and the Least Significant Difference (LSD) test were used to determine significant differences among treatments (α = 0.05).

## 3. Results and Discussion

### 3.1. Optimization of Parameters for Obtaining MEO-NE Using I-Optimal Design

On the one hand, Table 2 presents the experimental matrix, including 24 runs carried out according to the I-optimal design, along with the responses (experimental). The multiple linear regression analysis of the experimental data produced second-order polynomial models to predict the droplet size (*Y*_1_, nm), ζ-potential (*Y*_2_, mV), and DPPH inhibition (*Y*_3_, %) values of optimized MEO-NE, which are described by Equations (9), (10), and (11), respectively, as functions of the coded factors (*X*_1_, *X*_2_, *X*_3_, and *X*_4_) and their interactions for the proposed fit.

On the other hand, Table 3 presents the ANOVA for the linear, quadratic, and interactive terms of the independent variables for each response (droplet size, ζ-potential, and DPPH inhibition) in the optimized MEO-NE (F-value). The quality of the proposed models was investigated by analyzing the coefficients of determination (R^2^) and the F-values. The R^2^ values (0.9685, 0.9685, and 0.8942 for droplet size, ζ-potential, and DPPH inhibition, respectively) represent the proportion of experimental variability that could be explained by the statistical model (regression equation) within the studied region.

Droplet size (nm):

(9) $$\begin{array}{c} Y_{1}=300.20-25.78X_{1}-26.67X_{2}+15.36X_{3}+279.03X_{4}+21.58X_{1}X_{2}+\\ 58.55X_{1}X_{3}+0.5389X_{1}X_{4}-31.29X_{2}X_{3}-25.03X_{2}X_{4}+44.26X_{3}X_{4}+19.33X_{1}^{2}-\\ 18.48X_{2}^{2}+42.99X_{3}^{2} \end{array}$$

ζ-potential (mV):

(10) $$\begin{array}{c} Y_{2}=-24.44+0.8857X_{1}+0.5714X_{2}+0.5504X_{3}-4.17X_{4}+0.4313X_{1}X_{2}-\\ 0.2822X_{1}X_{3}-0.3428X_{1}X_{4}+1.52X_{2}X_{3}-0.2953X_{2}X_{4}-0.3511X_{3}X_{4}-\\ 0.7547X_{1}^{2}+0.3175X_{2}^{2}+1.83X_{3}^{2} \end{array}$$

*DPPH^●^* inhibition (%):

(11) $$\begin{array}{c} Y_{3}=83.68-0.2780X_{1}+1.18X_{2}+3.26X_{3}-6.68X_{4}-1.38X_{1}X_{2}+\\ 0.2983X_{1}X_{3}+1.43X_{1}X_{4}-2.45X_{2}X_{3}+2.35X_{2}X_{4}+1.45X_{3}X_{4} \end{array}$$

The goodness of fit was also confirmed by the lack-of-fit test, as indicated by the lack of significance (*p* > 0.05) for the three responses. Thus, there is no evidence of lack of fit for the models specified in Equations (8)–(10). All the values had a 95% confidence level. Residual analysis for the three proposed models (not included in this article) also confirms the goodness of fit to the experimental data. Given that the R^2^ values were ~1, there was no lack of fit, and the residuals met the statistical assumptions, it can be stated that the proposed models adequately describe the process and predict the behavior of the response variables with relative safety for preparing MEO-NE.

### 3.2. Characterization of Optimized MEO-NE

The highest desirability value (0.788) was obtained as a function of droplet size, ζ-potential, and *DPPH^●^* inhibition using an I-optimal design. The optimal conditions were 6%, 9 min, 5.24%, and Tween^®^ 80 for emulsifier concentration (X_1_), sonication time (X_2_), essential oil concentration (X_3_), and type of emulsifier (X_4_), respectively. Table 4 shows the predicted and experimental responses of MEO-NE stabilized with Tween 80, along with other characteristics (PDI, viscosity, and pH). Despite the emulsions stabilized with sapote gum achieving ζ-potential values very close to −30 mV, they exhibited the greatest droplet diameters (Table 3). Therefore, it was decided to prepare and characterize the films using the optimized MEO-NE formulation stabilized with Tween^®^ 80.

#### 3.2.1. Droplet Size, Polydispersity Index (*PDI*), ζ-Potential, *DPPH^●^* Inhibition, and *ABTS^●+^* Inhibition of MEO-NE Under Optimized Conditions

Tween^®^ 80 can form small droplets in emulsions during homogenization due to its molecular weight and structure [36]. Optimized MEO-NE, stabilized with Tween^®^ 80, showed reduced droplet size (Table 4), based on intensity-weighted distributions, indicating that this emulsifier adsorbed onto the droplet surface, which decreased the interfacial tension between the water and oil phases. In this regard, Chu et al. [24] reported that cinnamon essential oil nanoemulsions containing 6% Tween^®^ 80 and subjected to 10 min of ultrasound treatment showed the smallest droplet size (~60 nm) and the highest stability.

Gul et al. [37] suggested that PDI is a dimensionless measure of the droplet size distribution, ranging from 0.05 for monodispersed distributions to 0.7 for broad distributions. As seen in Table 5, PDI was sufficiently low according to the reports of Gul et al. [37] and Tastan et al. [38] for hazelnut protein-based clove essential oil nanoemulsions (0.277–0.478) and carvacrol essential oil nanoemulsions (0.23–0.42), respectively.

ζ-potential is a crucial factor in evaluating the stability of emulsions. A high absolute ζ-potential indicates strong electrostatic repulsion between particles, helping the emulsion or colloidal system remain stable and prevent flocculation and coalescence. ζ-potential value of ±30 mV is considered to be sufficient for emulsion systems to maintain such stability [39]. As shown in Table 4, the ζ-potential value (−15.0 mV) was negative due to the negative surface charge of the MEO droplets and the non-ionic Tween^®^ 80 used; regarding this, McClements [40] suggested that ζ-potential values between −10 mV and −20 mV could indicate short-term stability, but with a risk of instability under certain conditions. This result means that the obtained nanoemulsion was not stable, which was confirmed by an accelerated stability test using LUMiSizer^®^ equipment. Furthermore, the lowest droplet size of MEO-NE stabilized with Tween^®^ 80 did not show the highest stability; therefore, an alternative could be to mix this with a natural biopolymer, such as sapote gum and others.

The antioxidant assay of optimized MEO-NE, stabilized with Tween^®^ 80, titrating against *DPPH^●^* and *ABTS^●^^+^* radicals, is an essential indicator for evaluating the antioxidant capacity of substances in vitro (Wang et al. [41]). In this study, MEO-NE showed higher antioxidant activity. At the concentration of 5.24 g MEO/100 g, the antioxidant activity of MEO-NE reached 95.6% and 89.6% (Table 4) for *DPPH^●^* and *ABTS^●^^+^* radicals, respectively. This may be due to the high volatility of pure MEO, resulting in a shorter reaction time for its active components, such as those in the essential oil nanoemulsion. According to Wang et al. [41], the smaller droplet size of nanoemulsions gives more comprehensive contact with free radicals. These results indicate that the MEO-NE could be used as an easily accessible natural antioxidant additive in the food industry and in active films.

#### 3.2.2. Viscosity and pH of MEO-NE

Nanoemulsions exhibited Newtonian behavior with viscosities similar to those of pure water (Table 4), likely due to the low oil phase content. McClements [42] suggested that non-ionic emulsifiers tend to form more fluid, less viscous systems due to their simple molecular structure and lower tendency to form intermolecular crosslinks. This is because they can form swollen micelles with a small optimal radius, resulting in low interfacial tension and, therefore, lower system viscosity. This lower viscosity can be advantageous in applications that require higher fluidity and easy spreading.

The pH value (4.1) for the MEO-NE containing Tween^®^ 80 (Table 4) is characteristic of emulsions prepared with non-ionic emulsifiers, which maintain a slightly acidic environment. This pH is favorable for the stability of certain emulsions and for maintaining the integrity of the volatile compounds of the essential oil. An opposite result was reported by Moradi and Barati [43] (pH = 7–8) for nanoemulsions prepared with thyme, Shirazi thyme, and rosemary essential oils, using Tween^®^ 80 and/or sodium dodecyl sulfate as emulsifiers. Similar results were reported by Gorjian et al. [44] (pH = 7.20–7.64) for spearmint essential oil nanoemulsions, indicating their short and long-term physical and chemical stability. MEO contains active compounds, such as menthol and pulegone, that are highly pH-dependent [45]. At low pH values, the carboxyl groups of fatty acids are non-ionized and fat-soluble, allowing them to stabilize the emulsion. However, as pH increases, these carboxyl groups ionize and become water-soluble, thereby stabilizing oil-in-water emulsions [46].

#### 3.2.3. Creaming Stability Analysis of MEO-NE

**Figure 1:**
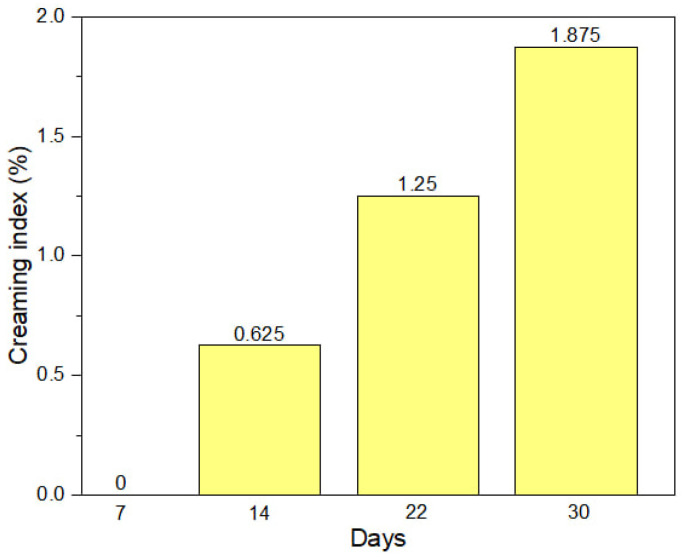
Evolution of *CI* of MEO-NE stabilized with Tween^®^ 80 under optimal conditions.

The creaming index of MEO-NE obtained was monitored at 7, 14, 22, and 30 days. The results show CI values of 0%, 0.625%, 1.250%, and 1.850%, respectively (Figure 1). Wu et al. [47] explained that Tween^®^ 80 effectively reduces surface tension, thereby favoring nanoemulsion stability and reducing creaming. The stabilizing effect of Tween^®^ 80 was independent of the measured physical properties, including zeta potential and apparent viscosity. This behavior is consistent with the observation of Tadros [48], who noted that emulsions stabilized with Tween^®^ 80 tend to exhibit lower creaming rates due to greater droplet size homogeneity and reduced coalescence.

#### 3.2.4. Accelerated Centrifugation Test of MEO-NE

**Figure 2:**
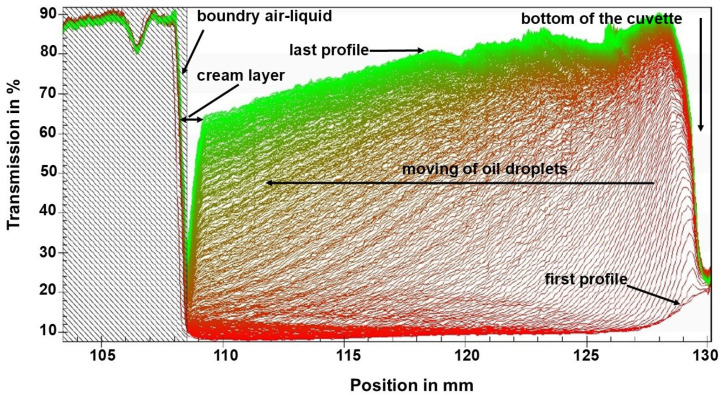
Transmission profiles of MEO-NE-stabilized with the addition of Tween^®^ 80 under optimal conditions obtained from LUMiSizer^®^ equipment.

Creaming stability is an essential indicator of an emulsion’s resistance to gravitational separation and can be evaluated via accelerated centrifugation tests. The physical stability of MEO-NE was analyzed using the LUMiSizer^®^ at 25 °C, a temperature simulating the storage temperature to which the emulsion could be subjected. According to Dammak & Sobral [49], high transmission indicates low droplet concentration, while low transmission indicates high droplet concentration. All the first profiles were located at the bottom (red transmission profiles), and the last ones were located at the top (green transmission profiles). The transmission profiles depicting the variation in oil droplet concentration over time inside the MEO-NE are shown in Figure 2. The 108.5 mm position corresponds to the meniscus air sample. As shown, the tested Tween 80 concentration (6%) destabilizes oil droplets, resulting in a polydisperse distribution (no sharp front) and creaming, as indicated by increased transmission at the top of the cell. As creaming progressed, the increased concentration of droplets in the meniscus region increased transmission. The separation of the MEO-NE reveals its instability (index = 0.817), as it exhibited a remarkable increase in transmission over time during the test. A similar pattern was reported by Dammak & Sobral [50] for rutin emulsions stabilized by 0.5% lecithin. This result confirmed that the nanoemulsion obtained was unstable. Given that antioxidant activity (DPPH inhibition) is considered a response for MEO-NE optimization, the levels of the optimized independent variables yielded ζ-potential values indicative of instability. Furthermore, the lowest droplet size of MEO-NE stabilized with Tween^®^ 80 did not correspond to the highest stability; therefore, an alternative could be to mix it with a natural biopolymer, such as sapote gum, and others.

#### 3.2.5. Atomic Force Microscopy of MEO-NE

**Figure 3:**
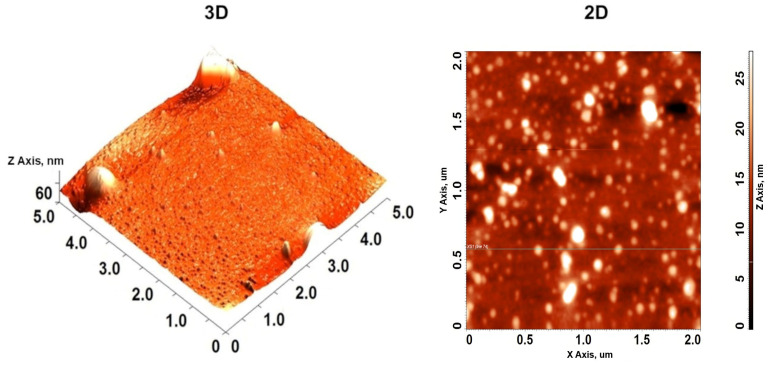
Atomic Force Micrography (AFM) images of 3D topography and 2D surface of MEO-NE under optimized conditions.

Typical 3-D and 2-D surface topographic AFM images of MEO-NE-stabilized with Tween^®^ 80 are illustrated in Figure 3. Optimized MEO-NE exhibited the presence of nanosized globular and smooth-surfaced droplets, which is consistent with the assessments obtained from the size analyzer by dynamic light scattering (DLS). The droplet size was less than 100 nm. The uniformity of droplet shape and size in MEO-NE indicates a well-distributed formulation. According to Galvao et al. [30], sample preparation or weak adsorption of droplets on a substrate’s surface can cause droplets to deform. Hence, it is essential to take precautions when analyzing these results.

### 3.3. Composite Films Characterization

#### 3.3.1. Film’s Appearance and Thickness

The addition of MEO-NE did not have a significant effect on composite films, which were slightly opaque. Nevertheless, the control and composite films had a similar good overall appearance, with no bubbles, scratches, or phase separation. The thickness of the composite films (Table 5) was maintained due to the same mass ratio of FFD to Petri dish area; thus, there were no significant differences (*p* < 0.05). A similar behavior was observed in irradiated nanocomposite tunta starch/tara gum films [23].

#### 3.3.2. Moisture Content (*MC*), Solubility in Water (*SW*), Water Vapor Permeability (*WVP*), and Contact Angle (*CA*)

The physicochemical properties of the composite films could be affected by their *MC*, i.e., it plays an essential role due to the high plasticizing effect of water molecules. Table 5 shows that, at concentrations of 5% and 10% MEO-NE, the *MC* of composite films significantly decreased (*p* < 0.05). The *MC* of composite films was around 12%; it could be suggested that the oil phase of MEO-NE was insufficient to affect the films’ hygroscopicity, which was defined by the biopolymeric matrix rather than by the incorporated lipophilic components. Nevertheless, it is crucial to carry out shelf-life studies of composite films to ensure the preservation of their mechanical and stability properties.

*SW* is a crucial film characteristic that can impact film integrity. The films F_1_ and F_2_, loaded with 5% and 10%, respectively, showed lower (*p* < 0.05) *SW* values than F_0_; this may be due to MEO’s insolubility in water (Table 5). Similar behavior was reported by Dalasso et al. [51] for active films produced using ginger oleoresin nanoemulsion (17.7–21.4%), by Pérez-Córdoba et al. [26] for gelatin-chitosan-based films loaded with nanoemulsions (43.1–48.9%), and by Fan et al. [52] for corn starch-based films loaded with clove essential oil nanoemulsion (29.01–34.58%). The decrease in *SW* values of composite films could be attributed to the interaction of polysaccharide molecules in nanoemulsions, which enhances molecular cross-linking and stereoscopic network formation in the composite starch-based films [52]. The low *MC* and *SW* values of composite films can improve the mechanical properties and broaden the application scope of starch-based films [52].

*WVP* is a critical property in films developed from hydrophilic materials [53]. *WVP* analysis revealed a significant decrease (*p* < 0.05) in the films loaded with MEO-NE compared with the control (Table 5). In well-structured starch films, the addition of hydrophobic compounds is often related to a decrease in *WVP* due to an increase in the matrix’s polarity [54]. The reduction in WPV values in biocomposite films could be due to irregular surfaces and tortuous channels introduced by the addition of an oil phase to the film matrix [55]. Similar patterns were reported by Flórez et al. [54] and Mutlu et al. [55].

The surface hydrophobicity of composite films is mainly affected by the types and concentrations of the film matrix and fillers. *CA* is a crucial parameter for evaluating the wettability and hydrophobicity of composite films [52]. As depicted in Table 5, the *CA* of composite films F_1_ and F_2_ gradually increased with the incorporation of MEO-NE (*p* < 0.05), and their values exceeded 65, indicating that these films were slightly more water-repellent than the control but still somewhat wettable. The addition of the MEO-NE slightly increased the contact angles of the films (F_1_ and F_2_), despite the presence of free hydrophilic surfactant molecules (Tween^®^ 80) in their formulations. According to Fan et al. [52], surfactants can decrease the contact angle of films, which is attributed to their capacity to adsorb at interfaces and lower surface or interfacial tension [55]. Therefore, the increased concentration of MEO-NE reduced the proportion of hydrophilic substances in the film matrix. These findings suggested that incorporating MEO-NE into starch films could enhance their *WVP*, which is valuable to food packaging.

#### 3.3.3. Mechanical Properties

Their mechanical properties evaluate the suitability of films. The tensile strength (*TS*) measures the maximum tensile stress that the films can withstand, and elongation at yield (*EY*) indicates the maximum change in length of the films before yielding [55]. Table 6 shows the mechanical properties of composite films. F_1_ films exhibited the highest *TS* values (30.71 MPa), indicating that they required greater stress to break. Young’s modulus (*YM*) indicated that F_0_ and F_2_ films displayed lower stiffness than F_1_ ones. Additionally, films loaded with MEO-NE showed higher *TS* than those without MEO-NE, which may be due to interactions among MEO-NE, chitosan, and starch. The homogenous dispersion of MEO-NE in the matrix could improve the tensile properties of the films. Regarding *EY* values, there was no significant difference between F_1_ and F_2_ films (*p >* 0.05); however, they were significantly different from F_0_ films. The incorporation of essential oils in excess could induce a plasticizing effect on the films, leading to discontinuities by weakening intermolecular interactions among polymeric chains [55].

#### 3.3.4. FTIR Analysis of Films

**Figure 4:**
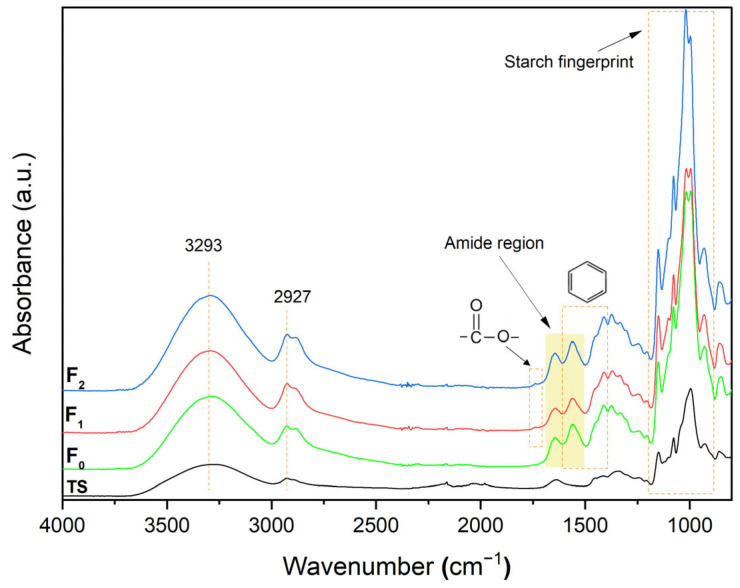
FT-IR spectra for composite films: TS: Tunta starch (Data from Martínez et al. [22]); F_0_: film without MEO-NE (Control); F_1_: film loaded with MEO-NE to achieve 5 g MEO/100 g polymers); F_2_ (film loaded with MEO-NE to reach 10 g MEO/100 g polymers).

**Figure 5:**
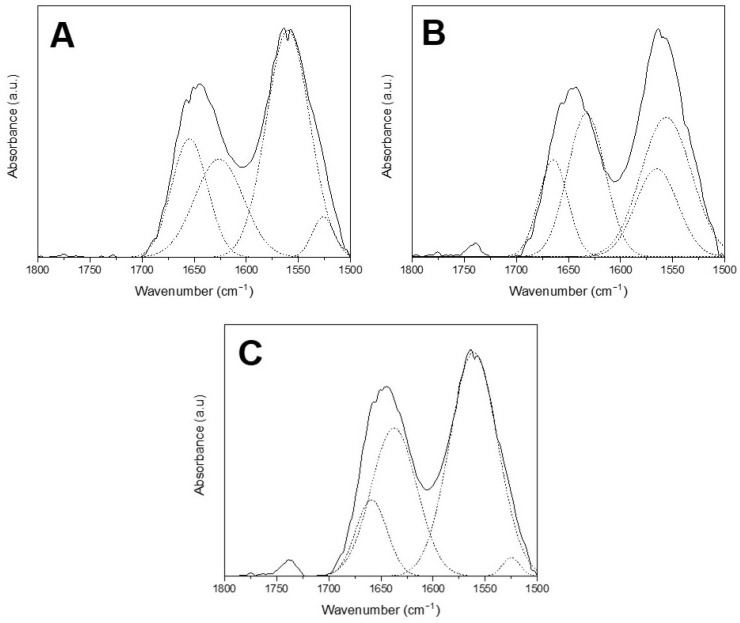
Deconvolution FT-IR spectra for composite films: (**A**) F_0_ films without MEO-NE (Control); (**B**) F_1_ films loaded with MEO-NE to achieve 5 g MEO/100 g polymers; (**C**) F_2_ films loaded with MEO-NE to achieve 10 g MEO/100 g polymers.

Figure 4 depicts the FTIR spectra and chemical structure of the developed composite films. The absorption band at 3293 cm^−1^ in the films corresponds to the stretching vibration of the hydroxyl group from polymers. This band is associated with the inter- and intramolecular OH groups of nearby starch molecules, indicating the formation of hydrogen bonds between the constituents in each film and its potential solubility in water [56]. At the same time, the absorption band at 2927 cm^−1^ corresponds to the stretching vibrations of C–H stretch [4] and C–H_2_ bonds of glycerol [56]. On the one hand, the amide I (C=O stretching) and amide II (N−H) regions, associated with chitosan presence, correspond to absorption bands at 1650 and 1560 cm^−1^, respectively [57]. On the other hand, tunta starch showed an absorption band at 1643 cm^−1^, assigned to bound water present in starch due to its hygroscopic nature [22]. The presence of Tween^®^ 80 was related to C=O bonds at 1738 cm^−1^, which is clearly observed in Figure 5; similar results were reported by Mao et al. [58] in carvacrol nanoemulsions. The bands in the region ranged from 900 to 1200 cm^−1^ and are shown to be sensitive to changes in starch structure, also known as the starch fingerprint [59]. According to Taylan et al. [60], the aromatic ring C=C skeleton for essential oils could be identified around 1573 cm^−1^. The differences in peak intensity and amplitude could correspond to rearrangement and conformational changes in the starch matrix due to interactions among components within MEO-NE and films [52].

Also, Figure 5 depicts the FTIR spectra deconvolution of the amide region for each film sample, and Table 7 shows the peaks detected by deconvolution, which evidence the changes in the region 1500–1700 cm^−1^.

#### 3.3.5. Antioxidant Activity

Active starch-based films containing MEO-NE at 5 and 10 g MEO/100 g polymers (F_1_ and F_2_, respectively) had a more antioxidant effect than films without MEO-NE (F_0_) (*p* < 0.05). The best inhibitory effects were seen at the 10 g MEO/100 g polymers, equal to 59.2% and 43.05% in *DPPH*^●^ and *ABTS*^●+^ scavenging assays, respectively (Table 8). This result is consistent with the reported high antioxidant capacity in films encapsulating phenolic compounds [26]. The control film showed the lowest radical-scavenging activity in the *DPPH*^●^ and *ABTS*^●+^ assays; therefore, the antioxidant activity of the developed films could be mainly due to the Chitosan content, which has a high antioxidant capacity. Among the analyzed films, the F_2_ films exhibited the highest antioxidant activity against both radicals. This could be due to a more open structure of the tunta starch-chitosan chains, affecting film integrity and resulting in higher solubility and concentration of the released active compounds, which in turn lead to higher antioxidant activity values [26]. Furthermore, the F_1_ and F_2_ films exhibited high free-radical-scavenging activity, probably because of the loading of the polyphenols and sesquiterpene-rich extract in the blend [23]. Additionally, this could be due to the contribution of the residual free amino groups of the Chitosan, which ionically react with free radicals, forming stable ammonium groups and macromolecular radicals.

#### 3.3.6. Disintegrability Test

**Figure 6:**
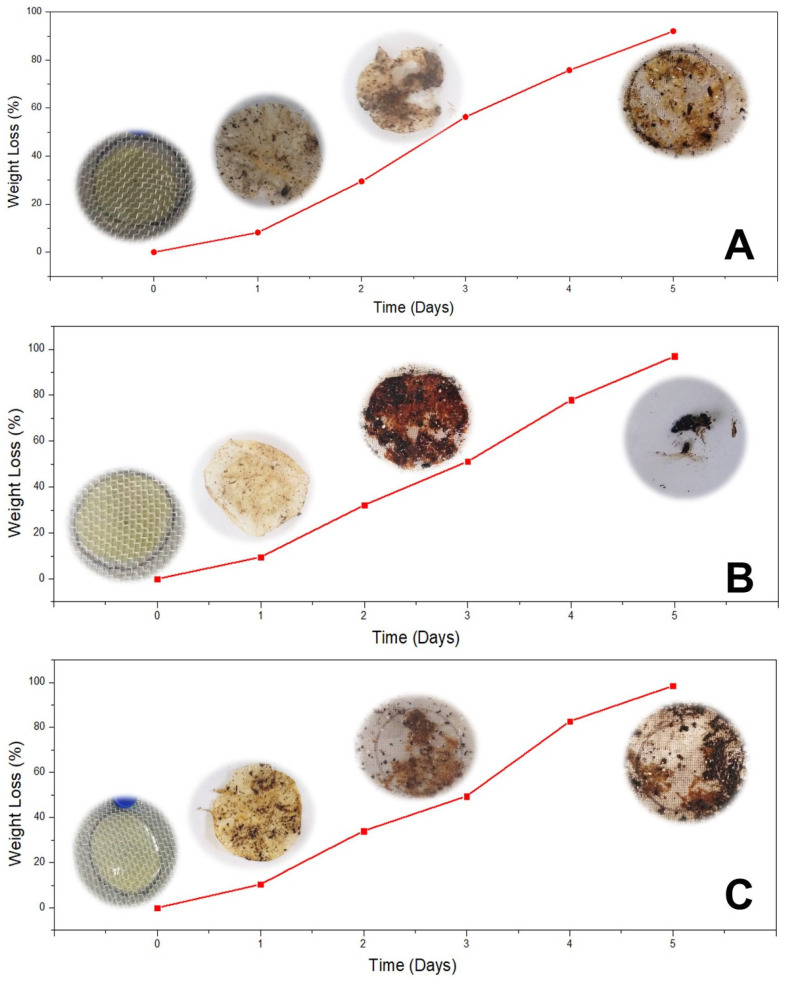
Disintegrability test results of (**A**) F_0_ (Control), (**B**) F_1_, and (**C**) F_2_ composite films in soil.

The disintegration of the films primarily depends on moisture and chemical structure, as evidenced by weight changes and surface microstructure. This disintegration occurs due to the action of enzymes and involves living soil microorganisms [61,62]. The compost soil used for the disintegrability test was reported by Pérez-Córdoba et al. [23]. In this work, F_1_ and F_2_ films exhibited higher weight losses (97% and 98%, respectively) after 5 days than the F_0_ (92%) ones. Indicating a positive disintegration process (Figure 6). According to the Standard ISO 20200:2015 [34], material is considered disintegrable when 90% of its mass is lost and the residues are below 2 mm within 90 days of the burial test. Similar results were reported by Bonilla and Sobral [63]. This is due to the relationship between the available moisture and the enzymes of soil compost microorganisms on the films [23]. The rapid disintegration of composite films could be attributed to soil moisture, which can easily penetrate the biopolymer intermolecular interactions, weakening their chains and facilitating hydrolysis by soil microorganisms [61,62]. Furthermore, due to intermolecular interactions and material performance, the F_0_ films (Control) were less disintegrable than the composite ones but still achieved 92% disintegration. Although starch provides a sustainable alternative to plastic, it is essential to evaluate the environmental impact of these films throughout their entire industrial life cycle —from production to use and disposal. It is also necessary to investigate whether these films release any components that could harm ecosystems.

## 4. Conclusions

In this study, optimized MEO-NE ultrasound-assisted stabilization with Tween^®^ 80 was incorporated into tunta starch-based active composite films using the casting method. Tuna starch dispersion, Chitosan solution, and MEO-NE were blended to obtain composite starch-based films (F_0_, F_1_, and F_2_), which were characterized using several techniques. The effects of the MEO on the properties of composite films were investigated. The incorporation of MEO-NE into starch composite films decreased *WVP*, which is valuable to food packaging. The incorporation of MEO-NE influenced the mechanical properties of films; *TS* and *YM* values for F_1_ and F_2_ films were higher than those of F_0_ films. Also, the antioxidant activity of the composite films increased due to the addition of MEO-NE. Finally, F_1_ and F_2_ films (loaded with MEO-NE) exhibited higher disintegration, achieving 98% disintegration in 5 days. The characteristics of these active composite starch-based films could be further improved to meet requirements for commercial applications in the food industry by optimizing film-forming formulation variables, such as NE concentration and the types and concentrations of plasticizers.

## Figures and Tables

**Table 1:** Levels at coded factor levels of independent variables used in the I-optimal design.

Independent Variable	Type	Levels	
		Minimum	Maximum
Emulsifier concentration (%, *w*/*w*)	Numeric	6	10
Essential oil concentration (%, *w*/*w*)	Numeric	3	6
Sonication time (min)	Numeric	9	15
Emulsifier	Categorical	Tween^®^ 80	Sapote gum

**Table 2:** I-optimal design matrix and responses obtained for the different nanoemulsion formulations in the study.

Run	Factors				Responses		
	X_1_	X_2_	X_3_	X_4_	Y_1_	Y_2_	Y_3_
1	9.68	12.0	4.44	Tween^®^ 80	13.96 ± 0.88	−21.10 ± 0.52	91.20 ± 0.54
2	10.00	9.0	3.00	Tween^®^ 80	11.89 ± 0.63	−18.06 ± 2.67	88.98 ± 0.49
3	6.00	9.0	3.87	Tween^®^ 80	89.44 ± 26.16	−22.40 ± 3.08	89.57 ± 0.37
4	10.00	9.0	4.93	Tween^®^ 80	17.69 ± 3.28	−24.23 ± 4.86	91.81 ± 0.38
5	8.30	9.0	4.04	Sapote gum	687.96 ± 15.40	−27.63 ± 0.21	70.97 ± 1.08
6	7.72	15.0	3.02	Tween^®^ 80	129.02 ± 9.18	−22.26 ± 4.18	88.35 ± 0.36
7	6.00	13.0	5.78	Tween^®^ 80	24.13 ± 3.12	−20.33 ± 0.45	93.13 ± 0.36
8	9.90	11.0	3.00	Sapote gum	414.46 ± 28.22	−26.80 ± 0.40	68.22 ± 0.33
9	6.10	15.0	4.65	Sapote gum	429.26 ± 41.97	−27.56 ± 1.09	73.12 ± 0.37
10	9.92	15.0	4.93	Sapote gum	579.23 ± 82.54	−27.16 ± 0.37	75.01 ± 0.60
11	6.08	11.0	3.00	Sapote gum	708.56 ± 237.43	−29.53 ± 0.15	70.35 ± 1.11
12	6.00	9.0	6.00	Sapote gum	746.13 ± 46.39	−28.60 ± 0.26	76.81 ± 2.37
13	7.92	11.0	3.00	Tween^®^ 80	12.98 ± 0.77	−16.46 ± 0.66	89.07 ± 0.87
14	9.68	12.0	4.44	Tween^®^ 80	16.23 ± 2.12	−17.03 ± 1.90	81.39 ± 1.61
15	8.30	9.0	4.04	Sapote gum	506.73 ± 130.58	−28.20 ± 0.17	73.37 ± 0.26
16	10.00	15.0	6.00	Tween^®^ 80	23.43 ± 3.47	−13.70 ± 0.80	86.53 ± 1.33
17	6.60	15.0	3.00	Sapote gum	608.83 ± 89.65	−28.53 ± 0.45	84.02 ± 1.45
18	10.00	14.0	3.09	Sapote gum	501.60 ± 16.77	−27.53 ± 0.21	80.85 ± 0.68
19	8.30	13.0	5.85	Sapote gum	650.80 ± 8.88	−27.90 ± 0.45	87.38 ± 0.29
20	9.68	12.0	4.44	Tween^®^ 80	13.69 ± 0.21	−19.06 ± 2.54	92.34 ± 0.48
21	8.28	9.0	6.00	Tween^®^ 80	20.09 ± 1.08	−18.40 ± 1.08	94.34 ± 0.08
22	6.00	13.0	5.78	Tween^®^ 80	31.40 ± 1.06	−18.30 ± 1.35	94.12 ± 3.27
23	10.00	9.0	6.00	Sapote gum	763.33 ± 78.37	−27.40 ± 0.17	82.72 ± 0.27
24	8.30	13.0	5.85	Sapote gum	691.53 ± 60.13	−27.76 ± 0.35	86.31 ± 0.28

Values expressed as mean ± standard deviation (n = 3). X_1_, Emulsifier concentration (%); X_2_, Sonication time (min); X_3_, Essential oil concentration (%); X_4_, Type of emulsifier; Y_1_, Droplet size (nm), Y_2_, ζ-potential (mV); Y_3_, DPPH inhibition (%).

**Table 3:** ANOVA of linear, quadratic, and interactive terms of MEO-NE obtention variables on responses (F-value).

Term	Droplet Size (nm)	ζ-Potential (mV)	DPPH Inhibition (%)
Model	30.29 **	7.66 *	5.67 *
*X*_1_—Emulsifier concentration (%)	1.77	2.23	0.0489
*X*_2_—Sonication time (min)	1.59	0.7830	0.7524
*X*_3_—Essential oil concentration (%)	0.5799	0.7969	6.27 *
*X*_4_—Type of emulsifier	310.84 **	74.27 **	42.54 **
*X*_1_×_2_	0.7518	0.3213	0.7236
*X*_1_ *X*_3_	5.04 *	0.1252	0.0325
*X*_1_ *X*_4_	0.0007	0.3182	1.23
*X*_2_ *X*_3_	1.29	3.27	2.14
*X*_2_ *X*_4_	1.41	0.2098	3.10
*X*_3_ *X*_4_	4.71	0.3174	1.24
*X*_1_^2^	0.2473	0.4038	
*X*_2_^2^	0.2323	0.0734	
*X*_3_^2^	1.37	2.65	
Lack of fit	2.08	3.73	1.79
R^2^	0.98	0.91	0.81

*X*_1_, Emulsifier concentration (%); *X*_2_, Sonication time (min); *X*_3_, Essential oil concentration (%); *X*_4_, Type of emulsifier. * *p* < 0.05. ** *p* < 0.01.

**Table 4:** Predicted and experimental responses (Droplet size, **ζ**-potential, and DPPH inhibition) of MEO-NE stabilized with Tween^®^ 80 under optimized conditions (highest desirability).

Characteristics	Predicted	Experimental
Droplet size (nm)	54.3	48.6 ± 2.5
ζ-potential (mV)	−22.1	−15.0 ± 1.17
*DPPH^●^* inhibition (%)	93.1	95.6 ± 0.08
*ABTS^●^^+^* inhibition (%)	--	89.6 ± 0.70
*PDI*	--	0.223 ± 0.00
Viscosity (mPa·s)	--	1 ± 0
pH	--	4.1 ± 0.01

Values expressed as mean ± standard deviation (n = 3).

**Table 5:** Physicochemical characterization of composite films.

Sample	Thickness (mm)	Moisture Content (%)	Solubility in Water (%)	*WVP* × 10^−10^ (g·m^−1^ s^−1^ Pa^−1^)	Contact Angle (°)
F_0_	0.112 ± 0.005 ^a^	13.43 ± 0.48 ^c^	72.53 ± 2.25 ^c^	1.17 ± 0.06 ^b^	67.88 ± 0.56 ^a^
F_1_	0.114 ± 0.007 ^a^	8.56 ± 0.36 ^a^	60.56 ± 0.57 ^b^	0.93 ± 0.05 ^a^	72.11 ± 0.80 ^b^
F_2_	0.114 ± 0.003 ^a^	9.52 ± 0.49 ^b^	45.33 ± 0.18 ^a^	0.89 ± 0.05 ^a^	75.76 ± 0.26 ^c^

Values expressed as mean ± standard deviation (n = 3). Means in the same row followed by different letters are significantly different (*p* < 0.05). F_0_: film without MEO-NE (Control); F_1_: film loaded with MEO-NE to achieve 5 g MEO/100 g polymers); F_2_ (film loaded with MEO-NE to reach 10 g MEO/100 g polymers).

**Table 6:** Tensile strength (*TS*), Elongation at break (*EY*), and Young’s modulus (*YM*) of composite films.

Sample	Tensile Strength (MPa)	Elongation at Yield (%)	Young’s Modulus (MPa)
F_0_	12.58 ± 0.05 ^a^	4.17 ± 0.06 ^b^	610.62 ± 13.07 ^a^
F_1_	30.71 ± 0.63 ^c^	3.78 ± 0.05 ^a^	1425.82 ± 10.92 ^c^
F_2_	15.75 ± 0.33 ^b^	3.59 ± 0.18 ^a^	786.45 ± 18.48 ^b^

Values expressed as mean ± standard deviation (n = 3). Means in the same row followed by different letters are significantly different (*p* < 0.05). F_0_: film without MEO-NE (Control); F_1_: film loaded with MEO-NE to achieve 5 g MEO/100 g polymers); F_2_ (film loaded with MEO-NE to achieve 10 g MEO/100 g polymers).

**Table 7:** List of peak frequencies of the deconvoluted bands in the region 1500–1700 cm^−1^ for the composite films.

Sample	F_0_	F_1_	F_2_
Peak 1	1654	1665	1659
Peak 2	1626	1633	1637
Peak 3	1560	1565	1561
Peak 4	1526	1556	1525

F_0_: films without MEO-NE (Control); F_1_: films loaded with MEO-NE to achieve 5 g MEO/100 g polymers; F_2_: films loaded with MEO-NE to reach 10 g MEO/100 g polymers.

**Table 8:** *DPPH^●^* and *ABTS^●^^+^* scavenging assays of composite starch-based films loaded with MEO-NE.

Sample	*DPPH^●^* (% Inhibition)	*ABTS^●^^+^* Radical (% Inhibition)
F_0_	9.8 ± 0.88 ^a^	13.92 ± 0.04 ^a^
F_1_	53.9 ± 1.49 ^b^	28.46 ± 2.11 ^b^
F_2_	59.2 ± 1.02 ^c^	43.05 ± 2.06 ^c^

Values expressed as mean ± standard deviation (n = 3). Means in the same row followed by different letters are significantly different (*p* < 0.05). F_0_: film without MEO-NE (Control); F_1_: film loaded with MEO-NE to achieve 5 g MEO/100 g polymers; F_2_: films loaded with MEO-NE to reach 10 g MEO/100 g polymers.

## Data Availability

The original contributions presented in this study are included in the article; further inquiries can be directed to the corresponding authors.
